# OpenADAM: an open source genome-wide association data management system for Affymetrix SNP arrays

**DOI:** 10.1186/1471-2164-9-636

**Published:** 2008-12-31

**Authors:** J MY Yeung, P C Sham, A SW Chan, S S Cherny

**Affiliations:** 1 Department of Psychiatry, The University of Hong Kong, 21 Sassoon Road, Pokfulam, Hong Kong, PR China; 2 Genome Research Centre, The University of Hong Kong, 21 Sassoon Road, Pokfulam, Hong Kong, PR China; 3 The State Key Laboratory of Brain and Cognitive Sciences, The University of Hong Kong, 21 Sassoon Road, Pokfulam, Hong Kong, PR China

## Abstract

**Background:**

Large scale genome-wide association studies have become popular since the introduction of high throughput genotyping platforms. Efficient management of the vast array of data generated poses many challenges.

**Description:**

We have developed an open source web-based data management system for the large amount of genotype data generated from the Affymetrix GeneChip^® ^Mapping Array and Affymetrix Genome-Wide Human SNP Array platforms. The database supports genotype calling using DM, BRLMM, BRLMM-P or Birdseed algorithms provided by the Affymetrix Power Tools. The genotype and corresponding pedigree data are stored in a relational database for efficient downstream data manipulation and analysis, such as calculation of allele and genotype frequencies, sample identity checking, and export of genotype data in various file formats for analysis using commonly-available software. A novel method for genotyping error estimation is implemented using linkage disequilibrium information from the HapMap project. All functionalities are accessible via a web-based user interface.

**Conclusion:**

OpenADAM provides an open source database system for management of Affymetrix genome-wide association SNP data.

## Background

Large scale genome-wide association studies (GWAS) have become popular since the introduction of high throughput genotyping platforms. Hundreds of thousands of single nucleotide polymorphisms (SNPs) can be accurately and rapidly genotyped at a reasonable cost. GWAS allow researchers to pinpoint the genetic variations that are associated with susceptibility to complex disorders, such as bipolar disorder, coronary artery disease, Crohn's disease, hypertension, rheumatoid arthritis, and diabetes [1]. Affymetrix produce microarray genotyping technologies commonly used in GWAS, such as the Affymetrix GeneChip^® ^platform, which includes the Mapping 500K array and the Affymetrix Genome-Wide Human SNP Array 5.0 and 6.0, assaying up to nearly 1,000,000 SNPs on a single microarray chip.

GeneChip^® ^Genotyping Analysis Software (Affymetrix, Santa Clara, California) and Genotyping Console (Affymetrix, Santa Clara, California) are provided by Affymetrix to automate SNP allele calling and export of data. However, the functionality provided is rather primitive and lacks necessary features such as genotype data format conversion, allele and genotype frequency calculation, and data quality checking. While many existing software packages for downstream analysis of genetic data are command line programs designed for UNIX or Linux, the software provided by Affymetrix employs a graphical user interface designed solely for Microsoft Windows. Additionally, there may be multiple ongoing GWAS in an institution where different users should have different data access privileges. The existing software provided by Affymetrix may not be able to cope with actual data management needs, as there is a gap between the work flow of genotype data management and downstream statistical analysis of GWAS. With a view to filling this gap, the open source Association Data Management System (OpenADAM) package was developed.

## Implementation

The OpenADAM software package has been designed to allow management and data quality checking of the large amount of genotype data from the Affymetrix microarray platforms. The command line programs for genotype calling provided in the Affymetrix Power Tools (APT) are embedded in a software framework and act as the starting point of the data pipeline. The raw intensities in the CEL files generated from Affymetrix chips are analyzed by the DM, BRLMM, BRLMM-P or Birdseed algorithms implemented in the APT and converted into genotype calls. The genotype call dataset is then parsed by scripts written in the PERL programming language and loaded into the MySQL relational database server. The use of MySQL as the storage engine allows efficient retrieval and query of genotype data for downstream analysis. A web-based user interface is written in the PHP programming language for use with an Apache web server. OpenADAM is open source and relies only on open source software and software freely available from Affymetrix. It also takes advantage of the security protections of MySQL and the SSL version of the Apache web server.

## Results and discussion

The web interface allows users to remotely manage genotype data from their own projects with simple mouse clicks via a web browser. Each user of the system is given a user name and password to login and perform various operations. Users can view the list of called chips and the corresponding call rates, verify that samples done on the Nsp and Sty 250K arrays of the 500K microarray chip sets are indeed from the same individual, and calculate genotype and allele frequencies. Moreover, the system supports export of genotype data in formats suitable for downstream analysis using the standard software packages PLINK [2], Merlin [3] and Haploview [4]. Filters can be applied in order to export genotype data on a subset of chips or SNPs for tailored analysis.

In addition, a novel method to estimate the genotyping error rate is implemented in OpenADAM, which may serve as a data quality control metric and an aid in the calculation of statistical power. The estimation is based on linkage disequilibrium (LD) information of SNP pairs provided by the International HapMap Project [5]. About 7000 SNP pairs in the Affymetrix GeneChip^® ^array sets were selected which satisfy the requirement that each SNP in a pair has minor allele frequencies of at least 0.1 and the pair is in perfect LD (*r*^2 ^= 1) in each of the African, European, Chinese, and Japanese populations in the HapMap [5] data. Under these specific conditions, any mismatch in genotype between the two SNPs of a pair is likely to be the result of a genotyping error. However, this could also mean that LD is in fact not perfect in a particular GWAS sample, as a result of employing a population different from those represented in HapMap. Additionally, LD may not even be perfect for some of those 7000 SNP pairs in the HapMap populations, but due to limited sample size, recombinants may not have been observed in HapMap. The proportion of mismatched SNP pairs, therefore, provides a lower-bound estimate of the genotyping error rate of each GeneChip^®^. Assuming that a mismatch occurs when and only when one of the two SNPs in the pair is miscalled, the estimated error rate (*E*) is obtained from the proportion of SNP pairs with mismatched genotypes (*M *= 2[*E *- *E*^2^]), which gives $E=\frac{1-\sqrt{1-2M}}{2}\approx \frac{M}{2}$ for small mismatch rates. This expression assumes that if there is an error in calling both SNPs of a given LD pair, there would be no mismatch (that is, both SNPs would be called with the same incorrect genotype). Given that having an error on both SNPs of an LD pair would be a very rare event and further given that if there is an error on both SNPs it is not likely it would be the same type of error for systematic reasons, this simplification shouldn't bias our estimate of error rate very much.

**Figure 1:**
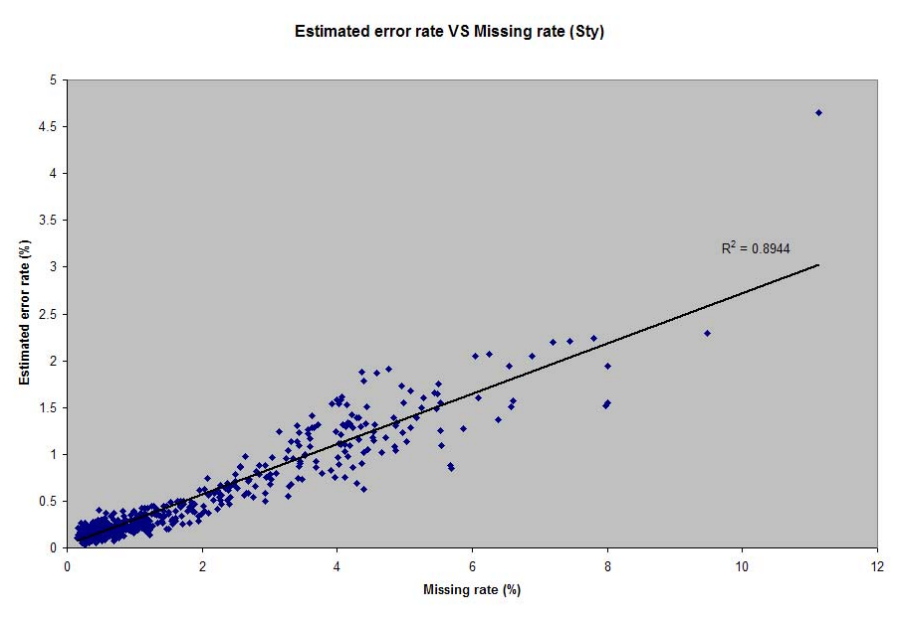
**Sample plot of estimated genotype error rate vs. mismatch rate for a sample of anonymous data genotyped using the Sty GeneChip^® ^from the 500K set**.

By plotting estimated chip error rate (*E*) versus chip call rate, for a sample of Affymetrix 500K Sty chips, as shown in Figure 1, we note the relationship between estimated error rate and call rate is very strong and linear (*r *= .95), which may aid in determining suitable cutoffs of call rate for discarding chips from analysis. From inspection of Figure 1 and other data (not shown), we further note little empirical evidence to justify the Affymetrix quality control threshold of 93% call rate in this particular sample, with perhaps a more liberal cutoff being equally acceptable, since error rate generally appears to be low (< 2.5%) for 90% call rate chips and not very different from chips of 93% call rate.

We further validated this approach to error estimation by correlating estimated error rate, from 14 pairs of samples which our lab had genotyped twice using the Affymetrix 5.0 platform, with error rate estimated using the LD method (averaged across each pair of chips done on an individual), and found the two estimates were reasonably highly correlated (*r *= .81). However, there was a strong tendency to overestimate error using the LD approach, as indicated by only 31% of LD-detected errors being confirmed by duplicate genotyping. Across our 28 chips where we estimated error based on the LD pairs, we found as many as 90% of errors confirmed for a chip with an estimated average error rate of .8% (and 45% confirmed for the chip with the highest estimated error rate of 1.99%), but none of the errors were confirmed for some chips with the lowest error rate estimates of .08–.28%. However, such low confirmation rates of LD-detected errors is somewhat misleading, given the substantial correlation (*r *= .68) between the LD-method estimated error rate and the percentage of confirmed errors, combined with the restricted range of error rates among these 28 chips. We suspect the method will be less biased for higher error rates (above 2%). Nonetheless, we caution the user that this approach appears to be only a very rough approximate estimate of the chip error rate. Further investigation of the approach is clearly needed to determine its validity.

## Conclusion

We have developed OpenADAM, a web-based data management system to streamline the workflow of the large amount of genotype data in genome-wide association studies using the Affymetrix GeneChip^® ^Mapping Array and Affymetrix Genome-Wide Human SNP Array platforms. This open source software will be a valuable resource for the research community performing GWAS.

## Availability and requirements

**Project name: **OpenADAM

**Project home page: **

**Operating system(s): **Platform independent

**Programming language: **MySQL, PERL, PHP

**Other requirements: **Apache webserver and Affymetrix Power Tools

**License: **GNU GPLv2

**Any restrictions to use by non-academics: **None

## Authors' contributions

PCS conceived of the software. JMYY, PCS, ASWC, and SSC designed the software. JMYY wrote the software code. JMYY and SSC wrote the manuscript and all authors revised and approved the final manuscript.

## References

[B1] The Wellcome Trust Case Control Consortium (2007). Genome-wide association study of 14,000 cases of seven common diseases and 3,000 shared controls. Nature.

[B2] Purcell S, Neale B, Todd-Brown K, Thomas L, Ferreira MAR, Bender D, Maller J, Sklar P, de Bakker PIW, Daly MJ, Sham PC (2007). PLINK: A Tool Set for Whole-Genome Association and Population-Based Linkage Analyses. Am J Hum Genet.

[B3] Abecasis GR, Cherny SS, Cookson WO, Cardon LR (2002). Merlin–rapid analysis of dense genetic maps using sparse gene flow trees. Nature Genetics.

[B4] Barrett J, Fry B, Maller J, Daly M (2005). Haploview: analysis and visualization of LD and haplotype maps. Bioinformatics.

[B5] The International HapMap Consortium (2007). A second generation human haplotype map of over 3.1 million SNPs. Nature.

